# The absence of intact polar lipid-derived GDGTs in marine waters dominated by Marine Group II: Implications for lipid biosynthesis in Archaea

**DOI:** 10.1038/s41598-019-57035-0

**Published:** 2020-01-15

**Authors:** Marc A. Besseling, Ellen C. Hopmans, Nicole J. Bale, Stefan Schouten, Jaap S. Sinninghe Damsté, Laura Villanueva

**Affiliations:** 1 NIOZ, Royal Netherlands Institute for Sea Research, Department of Marine Microbiology and Biogeochemistry, and Utrecht University., P.O. Box 59, NL-1790 AB Den Burg, The Netherlands; 2 Utrecht University, Faculty of Geosciences, P.O. Box 80.021, 3508 TA Utrecht, The Netherlands

**Keywords:** Lipids, Archaea, Biogeochemistry, Ocean sciences, Palaeoceanography

## Abstract

The marine pelagic archaeal community is dominated by three major groups, the marine group I (MGI) Thaumarchaeota, and the marine groups II and III (MGII and MGIII) Euryarchaeota. Studies of both MGI cultures and the environment have shown that the MGI core membrane lipids are predominantly composed of glycerol dibiphytanyl glycerol tetraether (GDGT) lipids and the diether lipid archaeol. However, there are no cultured representatives of MGII and III archaea and, therefore, both their membrane lipid composition and potential contribution to the marine archaeal lipid pool remain unknown. Here, we show that GDGTs present in suspended particulate matter of the (sub)surface waters of the North Atlantic Ocean and the coastal North Sea are derived from MGI archaea, and that MGII archaea do not significantly contribute to the pool of GDGTs and archaeol. This implies, in contrast to previous suggestions, that their lipids do not affect the widely used sea surface temperature proxy TEX_86_. These findings also indicate that MGII archaea are not able to produce any known archaeal lipids, implying that our understanding of the evolution of membrane lipid biosynthesis in Archaea is far from complete.

## Introduction

The dominance of archaeal communities in the marine pelagic ocean by marine group I (MGI) Thaumarchaeota and the marine group II and III (MGII and MGIII) Euryarchaeota has been well established by numerous studies^1–3^. It is known from culture and environmental studies that the Thaumarchaeota are capable of oxidizing ammonia^4,5^ and that some members are able to use urea as an alternative substrate^6^. The metabolism of MGII and MGIII archaea is thought to be (photo)heterotrophic and these Archaea are potentially able to degrade proteins, carbohydrates, fatty acids and other lipids^1,7–9^. However, these suggestions are based solely on metagenomic data since pure cultures of MGII and MGIII archaea have as yet not been obtained.

In the marine environment, archaeal membrane lipids are used as biomarkers for the presence of Archaea in microbial ecology studies^10,11^ but also in the paleotemperature proxy TEX_86_^12^, commonly used in paleoclimatological studies. The membrane lipid composition of the MGI archaea has been widely studied^13–20^ and found to include the diether lipid archaeol, glycerol dibiphytanyl glycerol tetraether (GDGT) lipids with zero to 4 cyclopentane moieties, and crenarchaeol, a GDGT with four cyclopentane moieties and a cyclohexane moiety, so far exclusively found in Thaumarchaeota^21^. In living and intact cells, GDGTs are mostly bound to polar head groups, termed intact polar lipid GDGTs (IPL-GDGTs). These occur mostly with sugar head groups such as monohexose (MH), dihexose (DH) and hexose phosphohexose (HPH)^13,16,18,19,22^.

Despite their importance in the marine water column as evidenced by metagenomic surveys^1,7–9^, it is not possible to directly determine the lipid membrane composition of MGII and MGIII archaea as pure cultures are lacking. A previous study based on a combination of metagenomics, sequencing of 16S rRNA gene amplicons and GDGT core lipid data in the North Pacific Subtropical Gyre has suggested that MGII archaea are producers of GDGTs, including crenarchaeol^23^. However, the low amount of extracted DNA and of archaeal gene reads together with the use of core lipid (CL)-GDGTs (which can also be attributed to cell debris) made the conclusions of this study ambiguous^24,25^. The absence of the gene encoding the enzyme required to synthesize the building block of archaeal GDGTs, i.e. glycerol-1-phosphate (G1P), in (meta)genomes of MGII and MGIII archaea strongly suggested that these Archaea are not able to synthesize ‘classical’ archaeal membrane lipids^26^. Furthermore, a recent study^27^ suggested that MGII archaea do not synthesize GDGTs with cyclopentane moieties. This is based on their genomes that lack the gene encoding the enzyme responsible for the internal cyclization reaction that results in the formation of cyclopentane and -hexane moieties. This would suggest that MGII archaea do not biosynthesize the GDGTs used in the TEX_86_ proxy. However, it is also unknown whether MGII archaea do make other typical archaeal lipids.

Here, we investigated the potential lipid composition of MGII archaea by characterizing the abundance and diversity of Archaea and composition of archaeal lipids in suspended particulate matter (SPM) of coastal North Sea water and the North Atlantic Ocean, which have previously been shown to contain high abundances of MGII and MGIII archaea^28,29^. We analyzed the abundance of the archaeal community by archaeal 16S rRNA gene quantitative polymerase chain reaction (qPCR), while the diversity of the archaeal community was analyzed with 16S rRNA gene amplicon sequencing. The abundance and composition of archaeal lipids were determined by Ultra High-Performance Liquid Chromatography coupled to High-Resolution Accurate-Mass/Mass Spectrometry (UHPLC-HRAM/MS), and the results were compared in order to constrain the importance of the three main archaeal pelagic groups in their contribution to the marine archaeal lipid pool.

## Results

We analyzed the ether lipid composition and archaeal diversity and abundance from suspended particulate matter (SPM) collected at depth intervals between 5–200 meters below sea level (mbsl) on glass-fiber filters with pore size 0.3 µm in the tropical North Atlantic (station 10 of Bale *et al*.^30^) and between 5–83 mbsl at four nearby stations using glass-fiber filters with pore size 0.7 µm. In addition, SPM of North Sea surface waters, collected in triplicate by sequential filtration through 0.7 µm and 0.3 µm filters, was analyzed for both lipids and DNA.

### Archaeal abundance and diversity

**Figure 1:**
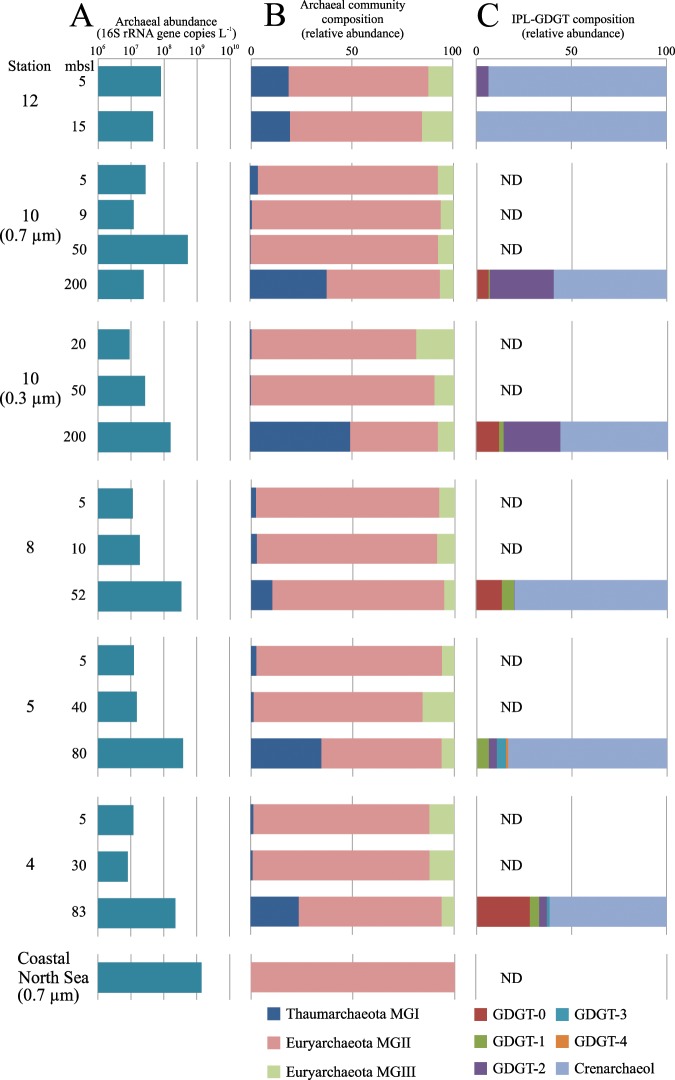
Archaeal abundance, community composition, and lipid distributions at six stations of the tropical North Atlantic Ocean at different water depths and North Sea coastal waters. (**A**) Total archaeal abundance determined by quantitative PCR and given as archaeal 16S rRNA gene copies L^−1^ of SPM. Numbers left of the bars indicate the sampling depth in meters below sea level (mbsl). (**B**) The archaeal community composition based on 16S rRNA gene amplicon sequencing. (**C**) The fractional abundances of IPL-GDGTs with various core lipids as specified in the legend. Abundances of IPLs containing the same core but with monohexose (MH), dihexose (DH) and hexose-phosphohexose (HPH) headgroups were summed assuming similar response factors. All stations were sampled with 0.7 µm glass-fiber filters, station 10 was also sampled with 0.3 µm glass-fiber filters. ND = not detected.

Total archaeal abundances in the tropical North Atlantic were low for the shallow waters (<50 mbsl), ranging between 0.8–8 × 10^7^ 16S rRNA gene copies L^−1^, compared to samples taken at greater depths (>50 mbsl), where they ranged between 0.2–5 × 10^8^ gene copies L^−1^ (Fig. 1A). The only exception was station 10 at 200 mbsl (0.7 µm), which had a lower amount of 16S rRNA gene copies L^−1^ than at 50 mbsl (Fig. 1A).

Various archaeal groups were detected in the SPM from the upper 200 m of the North Atlantic (Fig. 1B). At depths < 50 mbsl, the archaeal community consisted mainly of MGII archaea with relative abundances ranging between 65–93% of total archaeal 16S rRNA gene reads (Fig. 1B). At these depths, MGIII archaea were present in relative abundances of 6–18%, while the relative abundance of Thaumarchaeota is generally low (<4%, except for coastal station 12). At depths >50 mbsl, the relative abundance of MGI Thaumarchaeota ranged from 10–49%, while the MGII and III archaeal groups ranged from 43–84% and 5–8%, respectively.

The estimated abundances (obtained by multiplying their relative abundance given by the 16S rRNA gene amplicon sequencing by the total archaeal 16S rRNA gene copy abundance and assuming one 16S rRNA gene copy per MGII genome) of MGI archaea in North Atlantic SPM increased strongly at depths >50 mbsl, ranging between 1 × 10^5^ and 1 × 10^8^ 16S rRNA gene copies L^−1^ (Table 1). Abundances of MGII archaea varied between 7 × 10^6^ and 5 × 10^8^ 16S rRNA gene copies L^−1^ and increased with depth but not as noticeably as for the MGI archaea (Table 1). MGIII archaeal abundances were between 8 × 10^5^ and 3 × 10^7^ 16S rRNA gene copies L^−1^ (Table 1).

**Table 1:** North Atlantic Ocean sampling stations (cf Bale *et al*.^30^) and the coastal North Sea with sampling depth (mbsl), the pore size of the used glass-fiber filters, the amount of seawater (liter) filtered, the relative and estimated 16S rRNA gene abundances per liter of seawater filtered for the three main archaeal groups.

Station	Depth (mbsl)	Filter (pore size, µm)	Amount (l)	Relative abundance (%)			16S rRNA gene copies L^−1^		
				MGI	MGII	MGIII	MGI	MGII	MGIII
12	5	0.7	90	18.5	69.3	12.2	1 × 10^7^	5 × 10^7^	1 × 10^7^
	15	0.7	107	19.3	65.4	15.3	9 × 10^6^	3 × 10^7^	7 × 10^6^
10	5	0.7	271	3.9	88.4	7.7	1 × 10^6^	2 × 10^7^	2 × 10^6^
	9	0.7	89	1.0	92.7	6.3	1 × 10^5^	1 × 10^7^	8 × 10^5^
	50	0.7	361	0.3	92.0	7.7	2 × 10^6^	5 × 10^8^	4 × 10^7^
	200	0.7	399	37.7	55.6	6.7	9 × 10^6^	1 × 10^7^	2 × 10^6^
10	20	0.3	319	0.8	80.7	18.5	7 × 10^4^	7 × 10^6^	2 × 10^6^
	50	0.3	307	0.5	90.0	9.5	1 × 10^5^	2 × 10^7^	3 × 10^6^
	200	0.3	381	49.1	43.0	8.0	8 × 10^7^	7 × 10^7^	1 × 10^7^
8	5	0.7	234	2.4	89.9	7.7	3 × 10^5^	1 × 10^7^	9 × 10^5^
	10	0.7	247	2.8	88.6	8.7	5 × 10^5^	2 × 10^7^	2 × 10^6^
	52	0.7	381	10.4	84.4	5.2	3 × 10^7^	3 × 10^8^	2 × 10^7^
5	5	0.7	312	2.6	91.3	6.1	3 × 10^5^	1 × 10^7^	8 × 10^5^
	40	0.7	341	1.3	83.2	15.5	2 × 10^5^	1 × 10^7^	2 × 10^6^
	80	0.7	382	34.6	59.1	6.3	1 × 10^8^	2 × 10^8^	2 × 10^7^
4	5	0.7	306	1.4	86.4	12.2	2 × 10^5^	1 × 10^7^	1 × 10^6^
	30	0.7	331	1.0	86.9	12.1	8 × 10^4^	7 × 10^6^	1 × 10^6^
	83	0.7	380	23.7	70.2	6.0	5 × 10^7^	2 × 10^8^	1 × 10^7^
Coastal North Sea*	Surface	0.7	7.5–10	0.5	99.5	0.0	7 × 10^6^	1 × 10^9^	0 × 10^0^
	Surface	0.3	10	0.1	99.8	0.2	6 × 10^0^	7 × 10^3^	1 × 10^1^

The coastal North Sea suspended particulate matter filtered on the 0.3 µm filter was not included due to low biomass. *Coastal North Sea suspended particulate matter was sequentially filtered through the 0.7 µm glass-fiber filter followed by the filtration of the flow-through though the 0.3 µm glass-fiber filter.

The coastal North Sea waters contained 1 × 10^9^ archaeal 16S rRNA gene copies L^−1^ when filtered on a 0.7 µm glass-fiber filter (Fig. 1A). The archaeal 16S rRNA gene copy abundance was five orders of magnitude lower when the filtrate was subsequently filtered on a 0.3 µm glass-fiber filter (7 × 10^3^ archaeal 16S rRNA gene copies L^−1^; Table 1). In both cases, archaeal 16S rRNA gene reads were almost entirely (>99%) affiliated with MGII archaea (Fig. 1B; Table 1). The remaining archaeal 16S rRNA gene reads were derived of MGI archaea.

### Archaeal intact polar lipid diversity and distribution

None of the numerous, ca. 650 targeted archaeal IPLs (see inclusion list in Besseling *et al*.^31^) were detected within the SPM of the shallow waters (≤50 mbsl) of the North Atlantic (except for station 12) or the summer coastal North Sea SPM (0.7 µm filter) (Fig. 1C). In North Atlantic waters >50 mbsl, archaeal IPLs were detected (Fig. 1) with a dominance of hexose-phosphohexose (HPH) crenarchaeol (0–84% of the total detected IPL-GDGTs) and dihexose (DH) crenarchaeol (0–51%), and lower relative abundances of DH-GDGT-2 (0–33%) and HPH-GDGT-0 (0–28%) (Table 2). IPLs with crenarchaeol as their CL were the most abundant IPL-GDGTs in all cases (Fig. 1C). This was especially apparent at station 12, the station closest to the coast, where IPL-crenarchaeol dominated (94–100%; Fig. 1C).

**Table 2:** Fractional abundances (%) of all detected individual IPL-GDGT in the samples that contained IPL-GDGTs within the SPM from the tropical North Atlantic Ocean.

Station	Depth	GDGT-0		GDGT-1		GDGT-2			GDGT-3	GDGT-4	Crenarchaeol (and Cren′)		
	(mbsl)	MH	HPH	MH	HPH	MH	DH	HPH	DH	DH	MH	DH	HPH
12	5	0.0	0.0	0.0	0.0	0.0	6.4	0.0	0.0	0.0	9.9	0.0	83.7
	15	0.0	0.0	0.0	0.0	0.0	0.0	0.0	0.0	0.0	18.7	0.0	81.3
10 (0.7 µm)	200	2.1	4.1	0.7	0.0	0.7	33.0	0.0	0.0	0.0	8.1	51.3	0.0
10 (0.3 µm)	200	0.2	13.1	0.0	2.9	0.0	28.2	0.0	0.0	0.0	0.6	37.2	17.7
8	52	0.0	13.6	0.0	6.3	0.2	0.0	0.0	0.0	0.0	3.7	5.5	70.7
5	80	0.3	0.0	0.0	6.1	0.0	4.3	0.0	4.8	1.1	1.3	4.9	77.3
4	83	0.0	28.1	0.0	4.9	0.0	3.2	0.8	1.5	0.0	1.0	3.1	57.3

IPL-GDGTs were detected with the following headgroups: monohexose (MH), dihexose (DH) and hexose-phosphohexose (HPH).

**Figure 2:**
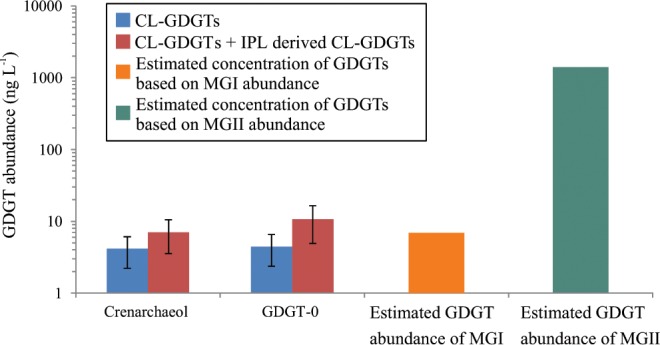
Mismatch between the cell abundance of MGII archaea and concentration of GDGT lipids in SPM of the coastal North Sea. SPM obtained with 0.7 um glass-fiber filters. The concentration of crenarchaeol, GDGT-0, the two most abundant GDGTs present in the form of core lipids (CL; blue bars) and present as CLs and IPLs (i.e. after acid hydrolysis of the Bligh Dyer extract; red bars) are shown. The indicated errors reflect the standard deviation of triplicate measurements. The estimated abundance of IPL-GDGTs based on the cell numbers derived from the measured gene copy numbers of MGI (orange bar) and MGII (green bar) archaea are shown for comparison. These estimations are based on the presence of 1 fg of IPL-GDGTs cell^−1^, an estimated abundance of 1.4 × 10^9^ MGII euryarchaeotal cells L^−1^ and 6.9 × 10^6^ MGI thaumarchaeotal cells L^−1^ (see text for details). Note the log-scale on the y-axis. The abundance of MGII archaea is at least two orders of magnitude too high to account for the GDGT concentration, whereas that of the MGI archaea is well in that range.

To investigate if there were IPL-GDGTs not covered by the ca. 650 targeted archaeal IPLs in our analysis, the IPLs of the North Sea SPM (0.7 µm filters), which is dominated by MGII archaea (1.4 × 10^9^ 16S rRNA gene copies L^−1^) and contains two orders of magnitude lower amounts of MGI archaea (6.9 × 10^6^ 16S rRNA gene copies L^−1^), were acid-hydrolyzed (which removes polar head groups from IPLs) to release IPL-derived GDGTs and archaeol (in triplicate). Analysis by UHPLC-MS using the selected ion monitoring mode showed that there was a small increase in the concentration after acid hydrolysis for GDGT-0 (from 4 ± 2 to 11 ± 6 ng L^−1^) and crenarchaeol (from 4 ± 2 to 7 ± 4 ng L^−1^) (Fig. 2). However, these small increases are statistically not significant (one-way ANOVA test; GDGT-0 (ρ = 0.15), crenarchaeol (ρ = 0.28)). Archaeol was barely detectable and concentrations were much lower than those of GDGTs, both before and after acid hydrolysis. This suggests that the MGII archaea, which are very abundant in these waters, do not produce IPLs with GDGTs or archaeol as core lipids and with head groups other than the targeted ones. To check whether the presence of archaeal core lipids other than GDGTs and archaeol could explain this discrepancy, we analyzed both the Bligh and Dyer total lipid extract and an acid-hydrolyzed lipid extract of the North Sea SPM with UHPLC Time-of-Flight Mass Spectrometry (ToFMS). Additional archaeal lipids detected were hydroxylated GDGTs (OH-GDGTs), glycerol dialkanol diethers (GDDs) and butanetriol dibiphytanyl glycerol tetraethers (BDGTs), though in very low abundance compared to the GDGTs (Fig. S5). Furthermore, no significant increase in concentration of these lipids after acid hydrolysis was observed, indicating that they do not occur in substantial amounts with an attached polar head group.

## Discussion

The archaeal community composition at station 10 at 200 mbsl in the North Atlantic and in the coastal North Sea was independent of the pore size of the filter used for filtration (Fig. 1B). Hence, the filter pore-size does not affect the archaeal community composition but only the amount of captured archaeal cells. MGII archaea were detected in high relative abundances in the shallow waters (0–50 mbsl) of the tropical North Atlantic Ocean (Fig. 1B). This is similar to other studies in which members of the MGII archaea were relatively more abundant near the surface compared to deeper waters^29,32,33^. Furthermore, the estimated MGII archaeal abundance in the North Atlantic ranged between 7 × 10^6^ and 5 × 10^8^ cells L^−1^, which is similar to previously reported values of the abundance of MGII archaea in the shallow waters of the North Atlantic Ocean (i.e. 3 × 10^7^ cells L^−1^)^29^. The MGII archaeal abundance in summer coastal North Sea was much higher, i.e. 1 × 10^9^ cells L^−1^, which is similar to previously reported MGII archaeal abundances and coincided within the known MGII archaea summer bloom at the same location^5^. Both the relative (Fig. 1) and absolute (Table 1) abundance of Thaumarchaeota MGI increased with depth in the tropical North Atlantic Ocean. This is in line with the reported niche preference of MGI archaea for subsurface waters (approximately 50–500 m depth)^10,23,32,34,35^. The MGI archaeal abundance in summer coastal North Sea was 7 × 10^6^ cells L^−1^, similar to earlier studies^19,28^ reporting MGI archaeal abundances of < 1 × 10^7^ cells L^−1^ in summer coastal North Sea SPM. Hence, the archaeal community composition and abundances in the SPM studied here compare well to those of previous studies and indicates an archaeal community composition in both surface waters (<50 mbsl) of the tropical North Atlantic and North Sea waters that almost entirely consists of MGII archaea.

**Figure 3:**
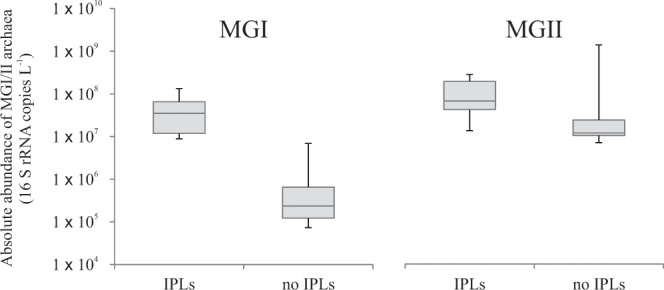
Estimates of abundances of Marine Thaumarchaeota (MGI) and Marine Euryarchaeota group II (MGII) (as cells L^−1^, based on archaeal 16S rRNA gene copies per liter) (median, first and third quartiles, whiskers depicting minimum and maximum values). Abundances in SPM from the tropical North Atlantic Ocean and North Sea coastal waters with detected intact polar lipid (IPL-) GDGTs (MH, DH, and HPH as headgroup) and in those in which IPL-GDGT were not detected.

IPL-GDGTs were only detected in SPM of the North Atlantic in which MGI archaea were present in substantial abundances (i.e. >9 × 10^6^ cells L^−1^; Fig. 3; Fig. S4). In contrast, in the shallow waters of the tropical North Atlantic and in the coastal water of the North Sea, the low abundances of MGI archaea (between 7 × 10^4^ and 7 × 10^6^ cells L^−1^; Fig. 1A) coincided with the lack of detection of IPL-GDGTs (Fig. 3; Fig. S4) and IPL-archaeol. Apparently below a certain MGI archaeal abundance (<~9 × 10^6^ cells L^−1^), the IPL-GDGTs and IPL-archaeol are below the detection limit of our IPL analysis method. These observations support previous reports that Thaumarchaeota are the dominant source of IPL-GDGTs in the ocean (cf. review by Schouten *et al*.^36^).

A different picture emerges for MGII archaea. At all stations in the North Atlantic and North Sea where the MGII archaeal abundance was >7 × 10^6^ cells L^−1^, i.e. the minimal abundance of MGI archaea at which their IPL-GDGTs can be detected, but when the MGI archaeal abundance was low (<7 × 10^6^), no IPL-GDGTs could be detected, nor any IPLs with an archaeol CL (Fig. 3; Table 1). Since the cell sizes and, therefore, the amount of IPLs per cell, are similar for MGI and MGII archaea, this strongly suggests that IPLs of MGII archaea are either not the same as commonly known archaeal IPLs or not detectable in our analytical window. To rule out the latter, acid hydrolysis was performed on SPM collected from the coastal North Sea (0.7 µm pore size). This only resulted in a minor and statistically insignificant increase of crenarchaeol and GDGT-0 compared to the CL-GDGTs already present (Fig. 2), which indicates the presence of only very low, if any, amounts of unaccounted IPL-GDGTs.

Further support for a predominant MGI archaeal source of GDGTs in the marine environment comes from an estimation of the IPL-GDGT concentration of MGI and MGII archaea, based on the amount of GDGTs expected to be present in MGI and MGII archaeal cells. MGI archaeal cells contain approximately 1 fg cell^−1^ of GDGTs based on culture and environmental studies (ranged from 1 fg cellular lipids cell^−1^, Sinninghe Damsté *et al*.^37^; 1.4 fg IPL-GDGTs cell^−1^ Lipp *et al*.^38^; 0.1–8.5 fg IPL-GDGTs cell^−1^, Huguet *et al*.^39^; 0.8–1.8 fg IPL-GDGTs cell^−1^ according to Elling *et al*.^13^). Thus, we would predict the presence of approximately 7 ng GDGTs per liter in coastal North Sea water derived from MGI archaea, in good agreement with the small and statistically insignificant, increase in concentration of crenarchaeol and GDGT-0 after acid hydrolysis (Fig. 2), which converts IPL-GDGTs into CL-GDGTs. The size of MGII archaeal cells is similar to that of MGI archaea^9^ and, therefore, they would be expected to contain approximately the same amount of IPL-GDGTs as estimated for MGI archaeal cells (i.e. 1 fg cell^−1^). Because of the orders of magnitude higher abundance of MGII archaeal cells in coastal North Sea SPM compared to MGI archaea, the expected concentration of IPL-GDGTs (or other archaeal lipids) attributed to MGII archaeal cells would be ca. 1,400 ng L^−1^. This is 2–3 orders-of-magnitudes higher than what is released after acid hydrolysis (Fig. 2). This mismatch indicates that if MGII archaea would produce IPL-GDGTs, acid hydrolysis would have indicated much higher concentrations of IPL-GDGTs than observed (Fig. 2).

Our data strongly suggests that MGII archaea do not produce known archaeal lipids such as GDGTs (or analogues thereof such as OH-GDGTs, BDGTs and GDD) or archaeol (diether lipids). Our results strongly contrast those of Lincoln *et al*.^23^, who stated that MGII archaea are able to synthesize archaeal GDGTs and, therefore, contribute substantially to the total archaeal GDGT pool, potentially affecting the paleotemperature proxy TEX_86_. Our results are in agreement with the recent study of Zeng *et al*.^27^ who suggested that MGII archaea might not synthesize GDGTs with cyclopentane and –hexane moieties based on the lack of cyclase coding genes in their genomes. Our study goes a step further as our results not only indicate the MGII archaea do not contribute to the GDGTs involved in the TEX_86_ proxy, but also do not seem to synthesize any other known archaeal membrane lipids.

Consequently, the question arises which lipids MGII archaea do produce. Phylogenomic analyses by Villanueva *et al*.^26^ of MGII archaeal genomes and metagenomes^7^ revealed that MGII archaea lack the gene coding for glycerol-1-phosphate dehydrogenase (G1PDH), the enzyme responsible for the formation of G1P in Archaea. This implies that they are not able to synthesize the G1P backbone found in archaeal membrane lipids, at least not by the classical biosynthetic pathway. However, MGII archaea still harbor other archaeal membrane lipid biosynthetic pathway genes such as geranylgeranylglyceryl phosphate synthase (GGGP) and digeranylgeranylglyceryl phosphate synthase (DGGGP), among others^26^. Therefore, they could potentially be capable of synthesizing isoprenoid-based ether lipids, but not with G1P as the glycerol building block, in contrast with other archaeal groups^40^. However, our current UHPLC-MS method would not be able to distinguish GDGTs of different glycerol stereochemistries.

Interestingly, MGII archaea also possess a putative fatty-acid biosynthetic pathway and putative homologs of the bacterial 1-acylglycerol-3-phosphate O-acyltransferase (PlsC; Villanueva *et al*.^26^) involved in esterifying fatty acids to glycerol-3-phosphate (G3P) in bacterial membrane lipids. MGII archaea could potentially produce a bacterial-like or a ‘chimeric’ lipid membrane, i.e. with a G3P backbone linked to di- or tetraether-linked isoprenoidal lipids, or lipids with one ether-linked isoprenoidal chain at position *sn*−1 and one ester-bound fatty acid at position *sn*-2 of the G3P backbone^26^. In fact, a mixed bacterial/archaeal lipid membrane could potentially favor the MGII archaea in variable environments. Caforio *et al*.^41^ recently demonstrated that the bacterium *E*. *coli*, when modified with archaeal/ether lipid biosynthetic genes, is able to synthesize a mixed lipid membrane, comprised of archaetidylglycerol (with a G1P backbone) alongside the bacterial lipids phosphatidylglycerol and phosphatidylethanolamine (both with a G3P backbone). The engineered *E*. *coli* with mixed membranes was more resistant to several chemical/physical stresses compared to the wild type *E*. *coli*^41^. However, careful inspection of the IPL composition as revealed by UHPLC-HRAM/MS did not reveal any unusual IPLs in high abundances or those which could be tentatively related to the hypothetical mixed lipids discussed above. A variety of other IPLs were detected in the North Atlantic (cf. Bale *et al*.^30^) and the coastal North Sea SPM. These are comprised of diacyl glycerides and common headgroups such as phosphatidylglycerol (PG), phosphatidylcholine (PC) and diacylglyceryl-carboxyhydroxymethylcholine (DGCC), among others (Fig. S2) and most likely have a bacterial or eukaryotic origin. This suggest that they either synthesize other non-archaeal IPLs commonly found in marine environments or more unusual IPLs which escape our analytical window. Clearly, the nature of the lipid membrane of MGII archaea will need to ultimately be confirmed by analyzing the membrane lipid composition of MGII archaea isolates.

## Conclusion

Analysis of North Atlantic Ocean and summer coastal North Sea SPM showed a dominance of MGII archaea with lower amounts of MGI and MGIII archaea. Only in SPM with a sufficient abundance of MGI archaea, we were able to detect known archaeal IPLs, but not in those with a dominance of MGII archaea such as summer coastal North Sea SPM containing abundant archaeal 16S rRNA gene reads attributed to MGII archaea. Acid hydrolysis followed by core lipid analysis ruled out the presence of intact polar archaeal lipids with unknown headgroup(s) in samples with dominant MGII archaeal populations. Based on our results, we conclude that it is unlikely that “planktonic Euryarchaeota are a significant source of archaeal tetraether lipids in the ocean”, as previously proposed by others^23^, or other known archaeal lipids. Therefore, the question remains as to which lipids MGII archaeal membranes do comprise. Further research focusing on the cultivation of the elusive MGII archaea and analysis of their membrane lipids are required to resolve this enigma.

## Methods

### Sampling and physicochemical analyses

Suspended particulate matter (SPM) was collected from the North Atlantic during the HCC cruise in September 2014 with *R/V* Pelagia^30^. SPM samples were taken with three McLane WTS-LV *in-situ* pumps (McLane Laboratories Inc., Falmouth, MA, USA). The pumps were deployed at various depths and locations (station numbers are according to Bale *et al*.^30^), i.e. between 5–200 meters below sea level (mbsl) at station 10 (6°41.04′N; 47°29.25′W), as well as from 5–83 mbsl at four nearby stations (station 12; 6°4.04′N; 52°27.69′W, station 8; 6°29.24′N; 45°26.98′W, station 5; 10°49.74′N; 40°28.20′W and station 4; 12°24.42′N; 38°30.12′W) (Fig. S1). SPM was filtered onto pre-ashed glass-fiber filters (0.7 µm nominal pore sizes, GF/F, 142 mm in diameter, Pall Corporation, Port Washington, NY) at all stations. Station 10 was also filtered on pre-ashed glass-fiber filters with 0.3 µm nominal pore sizes (GF75; 142 mm in diameter, Avantec, Japan). Between 89 and 399 liters of seawater were filtered (Table 1). Physical data and dissolved inorganic nutrients are reported in Bale *et al*.^30^). Coastal North Sea SPM was collected in July 2015 at high tide from the NIOZ jetty (53°0.1′N; 4°47.2′E). Coastal North Sea water (ranging around 7.5–10 liters) was filtered in triplicate through 0.7 µm and the filtrate subsequently on 0.3 µm pore size glass-fiber filters (Whatman, 142 mm in diameter). For all sampling locations, the filters were both used for lipid and nucleic acid analyses and were immediately stored at −80 °C after sampling.

### IPL extraction and analysis

Total lipids were extracted from freeze-dried glass-fiber filters using a modified Bligh and Dyer method^42^ as previously described earlier^43^. C_16_-PAF (1-O-hexadecyl-2-acetoyl-sn-glycero-3-phosphocholine) was added to the extracts as an internal standard. The extracts including the internal standard were dried under a stream of nitrogen, re-dissolved in known quantities of injection solvent (hexane:isopropanol:H_2_O 718:271:10 v/v/v) and filtered through a 0.45 µm, 4 mm-diameter True Regenerated Cellulose syringe filter (Grace Davison, Columbia, MD, USA).

IPLs were analyzed using UHPLC-HRAM/MS, designed for the analysis of a wide range of intact polar lipids. An Ultimate 3000 RS UHPLC, equipped with thermostated auto-injector and column oven, coupled to a Q Exactive Orbitrap MS with Ion Max source with heated electrospray ionization (HESI) probe (Thermo Fisher Scientific, Waltham, MA), was used. Separation was achieved on a YMC-Triart Diol-HILIC column (150 × 2.0 mm, 1.9 µm particles, pore size 12 nm; YMC Co., Ltd, Kyoto, Japan) with a guard column of the same material (10 × 2.1 mm) maintained at 30 °C. The following elution program was used with a flow rate of 0.2 mL min^−1^: 100% A for 5 min, followed by a linear gradient to 66% A: 34% B in 20 min, maintained for 15 min, followed by a linear gradient to 40% A: 60% B in 15 min, followed by a linear gradient to 30% A: 70% B in 10 min, where A = hexane/2-propanol/formic acid/14.8 M NH_3_aq (79:20:0.12:0.04 [v/v/v/v]) and B = 2-propanol/water/formic acid/14.8 M NH_3_aq (88:10:0.12:0.04 [v/v/v/v]). The samples from the North Atlantic (0.7 µm pore-size glass-fiber filters) were run with heptane instead of hexane in the mobile phase. Total run time was 70 min with a re-equilibration period of 20 min in between runs. HESI settings were as follows: sheath gas (N_2_) pressure 35 (arbitrary units), auxiliary gas (N_2_) pressure 10 (arbitrary units), auxiliary gas (N_2_) T 50 °C, sweep gas (N_2_) pressure 10 (arbitrary units), spray voltage 4.0 kV (positive ion ESI), capillary temperature 275 °C, S-Lens 70 V. IPLs were analyzed with a mass range of *m/z* 375 to 2000 (resolving power 70,000 at *m/z* 200), followed by data dependent MS^2^ (resolving power 17,500 ppm at *m/z* 200), in which the ten most abundant masses in the mass spectrum (with the exclusion of isotope peaks) were fragmented (stepped normalized collision energy 15, 22.5, 30; isolation window 1.0 *m/z*). A dynamic exclusion window of 6 s was used as well as an inclusion list with a mass tolerance of 3 ppm to target specific compounds (see Besseling *et al*.^31^ for the list of targeted compounds). The Q Exactive Orbitrap MS was calibrated within a mass accuracy range of 1 using the Thermo Scientific Pierce LTQ Velos ESI Positive Ion Calibration Solution (containing a mixture of caffeine, MRFA, Ultramark 1621, and N-butylamine in an acetonitrile-methanol-acetic acid solution).

Peak areas for each individual IPL were determined by integrating the combined mass chromatogram (within 3 ppm) of the monoisotopic and first isotope peak of all relevant adducts formed (protonated, ammoniated and/or sodiated adducts may be formed in different proportions depending on the type of IPL). C_16_-PAF was used as internal standard to continuously monitor MS performance and to assess matrix effects. Reported peak areas were corrected for these effects. Absolute quantification of IPL GDGTs was not possible due to a lack of authentic standards. Peak areas were not corrected for possible differences in response factors between the various classes of IPL-crenarchaeol. IPLs with crenarchaeol or its isomer(s) as its core lipid, but with the same headgroup, co-elute with the chromatographic system used here and any peak area reported for a crenarchaeol IPL thus represents the sum of both crenarchaeol and its isomer(s).

### Analysis of GDGTs and IPL-derived GDGTs

The Bligh and Dyer extracts from three separate 0.7 µm pore-size glass-fiber filters obtained from coastal North Sea water were analyzed for their CL-GDGTs and IPL-derived GDGTs. The extracts from the North Sea were dried under nitrogen and hydrolyzed separately with 1.5 M HCl in methanol by reflux at 130 °C for 2 h to remove the headgroups from the IPL-GDGTs and release the CL-GDGTs. The pH was adjusted to 7 by adding 2 M KOH/MeOH (1:1 v/v) and, after addition of water to a final 1:1 (v/v) ratio of H_2_O-MeOH, extracted three times with dichloromethane (DCM). The DCM fractions were collected and dried over sodium sulfate. The extracts containing IPL-derived CL-GDGTs and CL-GDGTs in case of the acid-hydrolyzed extracts or only CL-GDGTs in case of the not hydrolyzed extracts, were dried under N_2_ and dissolved in hexane–2-propanol (99:1, vol/vol) and filtered over a 0.45-µm polytetrafluoroethylene filter. Extracts were analyzed by UHPLC–atmospheric pressure chemical ionization (APCI) MS for archaeol and GDGTs, according to Hopmans *et al*.^44^, with some modifications. Briefly, analysis was performed on an Agilent 1260 UHPLC coupled to a 6130 quadrupole MSD in selected ion monitoring (SIM) mode. This allowed the detection of GDGTs with 0 to 4 cyclopentane moieties, crenarchaeol and is isomer as well as archaeol. Separation was achieved on two UHPLC silica columns (BEH HILIC columns, 2.1 × 150 mm, 1.7 µm; Waters) in series, fitted with a 2.1 × 5 mm pre-column of the same material (Waters) and maintained at 30 °C. Archaeol and GDGTs were eluted isocratically for 10 min with 10% B, followed by a linear gradient to 18% B in 20 min, then a linear gradient to 100% B in 20 min, where A is hexane and B is hexane: isopropanol (9:1). Flow rate was 0.2 ml/min. Total run time is 61 min with a 20 min re-equilibration. Source settings were identical to Schouten *et al*.^45^. Typical injection volume was 10 µl of a 1 mg/ml solution (weighted dried Bligh and Dyer extract dissolved in hexane:isopropanol (99:1, v/v ratio). The *m/z* values of the protonated molecules of archaeol and isoprenoid GDGTs were monitored. GDGTs were quantified by adding a C_46_ GTGT internal standard^46^. A response factor derived from an archaeol:GDGT-0 standard (1:1, wt:wt) was used to correct for the difference in ionization between archaeol and GDGTs.

The Bligh and Dyer extract and the acid hydrolyzed Bligh and Dyer extract were also analyzed using ultra high-performance liquid chromatography coupled to positive ion atmospheric pressure chemical ionization/Time-of-Flight mass spectrometry (UHPLC-APCI/ToFMS) on an Agilent 1290 Infinity II UHPLC, equipped with automatic injector, coupled to a 6230 Agilent TOF MS and Mass Hunter software. This additional analysis was performed to detect other ether lipids that were not included in our SIM method on the 6130 quadrupole MSD. Separation of the ether lipids was achieved according to Hopmans *et al*.^44^ with some modifications using two silica BEH HILIC columns in series (2.1 × 150 mm, 1.7 µm; Waters) at a temperature of 25 °C. The injection volume was 10 µL. Compounds were isocratically eluted with 90% A and 10% B for the first 10 min, followed by a gradient to 18% B in 15 min, a gradient to 30% B in 25 min and a linear gradient to 100% B in 30 min. A = hexane and B = hexane/isopropanol (9:1, v/v) and the flow rate was 0.2 mL/min. The conditions for the APCI source were identical to Schouten *et al*.^45^ and Hopmans *et al*.^44^. In addition, the fragmentor was set at 300 V. The ToFMS was operated in extended dynamic range mode (2 GHz) with a scan rate of 2 Hz. We assessed archaeal lipid distributions by monitoring *m/z* 600 to 1400. Ether lipids were identified by searching within 10 ppm mass accuracy for relevant [M + H]^+^ signals.

### Nucleic acids extraction and quantitative PCR (qPCR) analyses

DNA was extracted from the glass-fiber filters with the RNA PowerSoil® Total Isolation Kit plus the DNA elution accessory (Mo Bio Laboratories, Carlsbad, CA). 1/16^th^ of the filter was used of the 0.7 µm filters (North Atlantic Ocean and coastal North Sea samples) and 1/8^th^ of the filter of the 0.3 µm filters (North Atlantic Ocean, station 10). Concentration of DNA was quantified by Qubit fluorometric quantitation (ThermoFisher Scientific). Quantification of archaeal 16S rRNA gene copies were estimated by qPCR by using the primers Parch519F and ARC915R (archaeal 16S rRNA gene) as previously described by Pitcher *et al*.^10^. For details on the qPCR conditions, efficiency and R^2^ of the qPCR assays see Table S1.

### 16S rRNA gene amplicon sequencing, analysis, and phylogeny

PCR reactions were performed with the universal, Bacteria and Archaea, primers 515F-Y (5′-GTG YCA GCM GCC GCG GTA A-3′)^47^ and 806RB (5′-GGA CTA CNV GGG TWT CTA AT-3′)^48^ amplifying the V4 region of the 16S rRNA gene. Sequencing was done by the Utrecht Sequencing Facility (www.useq.nl) on a MiSeq platform (Illumina, CA, USA) 2 × 250 bp, using the MiSeq Reagent Kit v2 (Illumina). The 16S rRNA gene amplicon sequences were analyzed by Cascabel^49^, an amplicon sequence data analysis pipeline based on Snakemake^50^. Briefly, quality-filtered paired-end reads were assembled using PEAR (v0.9.8)^51^. Quality-filtered with a minimum quality score of 20 (Phred quality score), length between 250–350 bp, and allowing maximum two errors in the barcode sequence. The operational taxonomic units were picked based on a 97% similarity using UCLUST^52^ with pick_otus.py using QIIME^53^. Taxonomy was assigned with assign_taxonomy.py within QIIME, using UCLUST and based on the SILVA database version 128^52,53^. The phylogenetic affiliation of the partial archaeal 16S rRNA gene sequences was compared to release 123 of the SILVA NR SSU Ref database (http://www.arb-silva.de/; Quast *et al*.^40^) using the ARB software package^54^. Sequences were added to the reference tree supplied by the Silva database using the ARB Parsimony tool. Affiliation of any 16S rRNA gene sequences to a given subgroup was done assuming a similarity cutoff of ≥85%.

16S rRNA gene amplicon reads counts per sample are listed in Table S1. Rarefaction curves, indicating the OTU richness per sample (based on the 16S rRNA gene amplicon sequences, both bacterial and archaeal OTUs), are shown in Fig. S3.

Abundances of the three archaeal marine groups were estimated by multiplying archaeal copy number (16S rRNA gene copies L^−1^) by the relative abundance of the marine group (in % of all archaeal reads), assuming one copy of 16S rRNA gene per genome^55^.

## Supplementary information

Supplementary tables and figures.
