# Supplementary material for: The absence of intact polar lipid-derived GDGTs in marine waters dominated by Marine Group II: Implications for lipid biosynthesis in Archaea
Source: Sci Rep. 2020 Jan 15;10:294. doi: 10.1038/s41598-019-57035-0 (PMC6962369; doi:10.1038/s41598-019-57035-0)
Supplement: Supplementary file 1 — Supplementary tables and figures. [file 41598_2019_57035_MOESM1_ESM.pdf]

TITLE

The absence of intact polar lipid-derived GDGTs in marine waters dominated by Marine Group II: Implications for lipid biosynthesis in Archaea.

AUTHORS

Marc A. Besseling[1*], Ellen C. Hopmans[1], Nicole J. Bale[1], Stefan Schouten[1,2], Jaap S. Sinninghe Damsté[1,2] and Laura Villanueva[1]

AFFILIATIONS

[1]NIOZ, Royal Netherlands Institute for Sea Research, Department of Marine Microbiology and Biogeochemistry, and Utrecht University. P.O. Box 59, NL-1790 AB Den Burg, The Netherlands.

[2]Utrecht University, Faculty of Geosciences, P.O. Box 80.021, 3508 TA Utrecht, The Netherlands

CORRESPONDING AUTHOR

*e-mail: marc.besseling@nioz.com

KEYWORDS

Archaea, Marine Group II Euryarchaeota, Thaumarchaeota, intact polar lipids (IPLs), glycerol dibiphytanyl glycerol tetraether lipids (GDGTs), North Atlantic Ocean, coastal North Sea.

Supplementary tables and figures

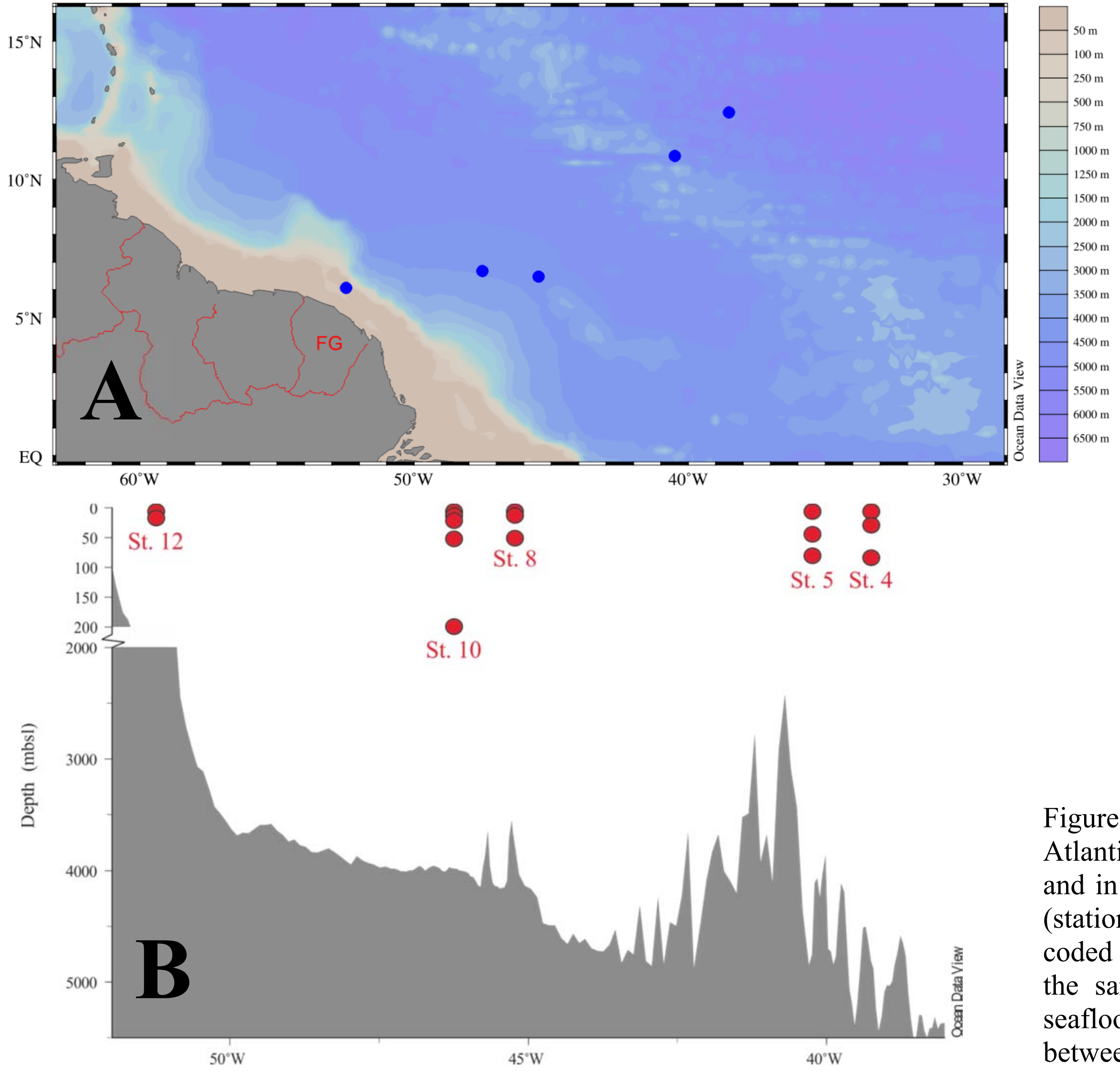

Figure S1**.** Details of the sampling in the tropical Northern Atlantic Ocean close to the coast of French Guiana (FG) and in the open ocean. (A) Map of the sampling locations (stations numbered and marked with blue dots). Color coded bathymetry scale on the right. (B) Depth profile of the sampled stations with the sampling depths and the seafloor profile. Note that the depth axis is cut off in between 200 and 2000 mbsl.

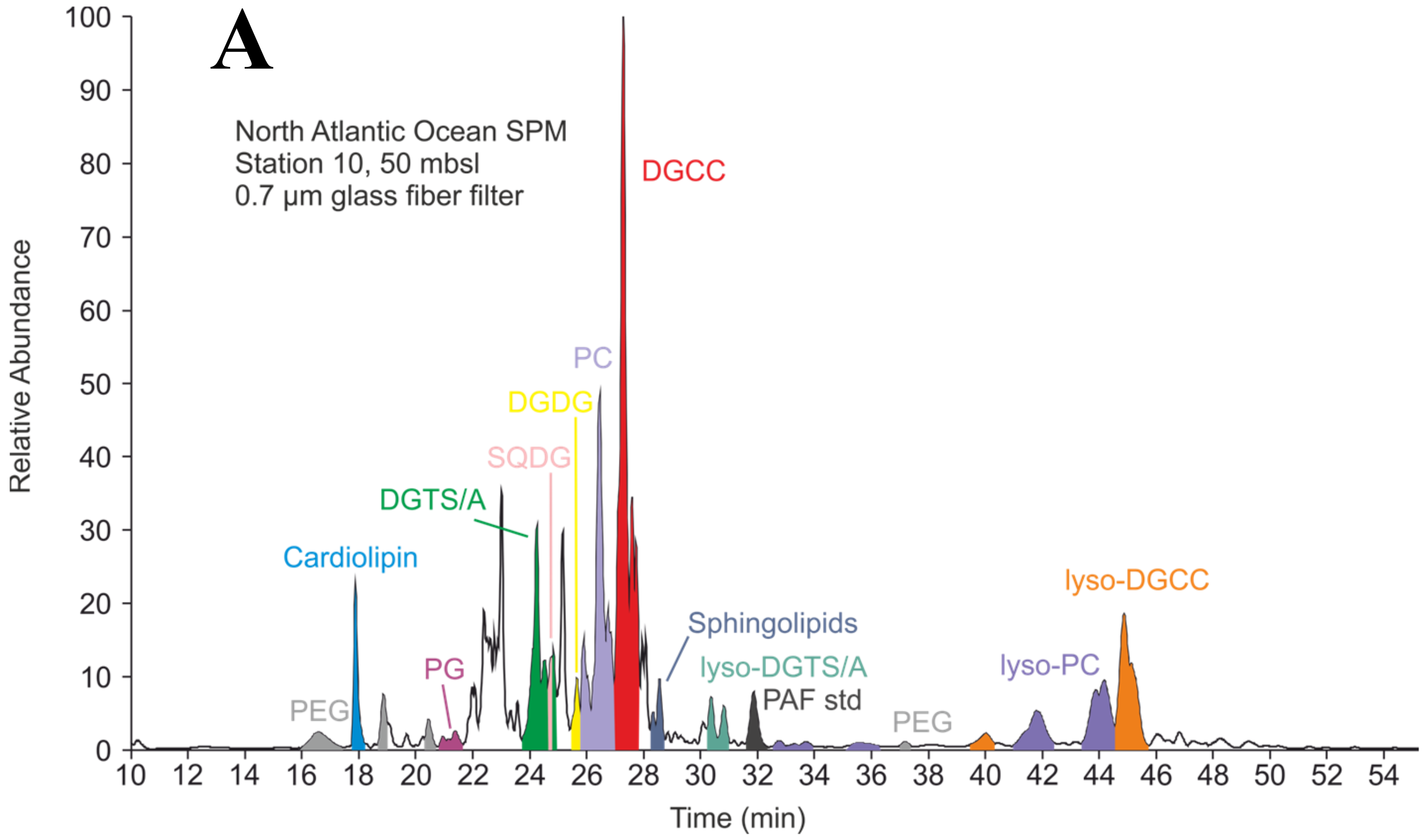
A
North Atlantic Ocean SPM
Station 10, 50 mbsl
0.7 µm glass fiber filter
Relative Abundance
Time (min)
DGCC
PC
DGDG
SQDG
DGTS/A
Cardiolipin
PG
PEG
Sphingolipids
lyso-DGTS/A
PAF std
PEG
lyso-PC
lyso-DGCC
0
10
20
30
40
50
60
70
80
90
100
10
12
14
16
18
20
22
24
26
28
30
32
34
36
38
40
42
44
46
48
50
52
54

Figure S2A

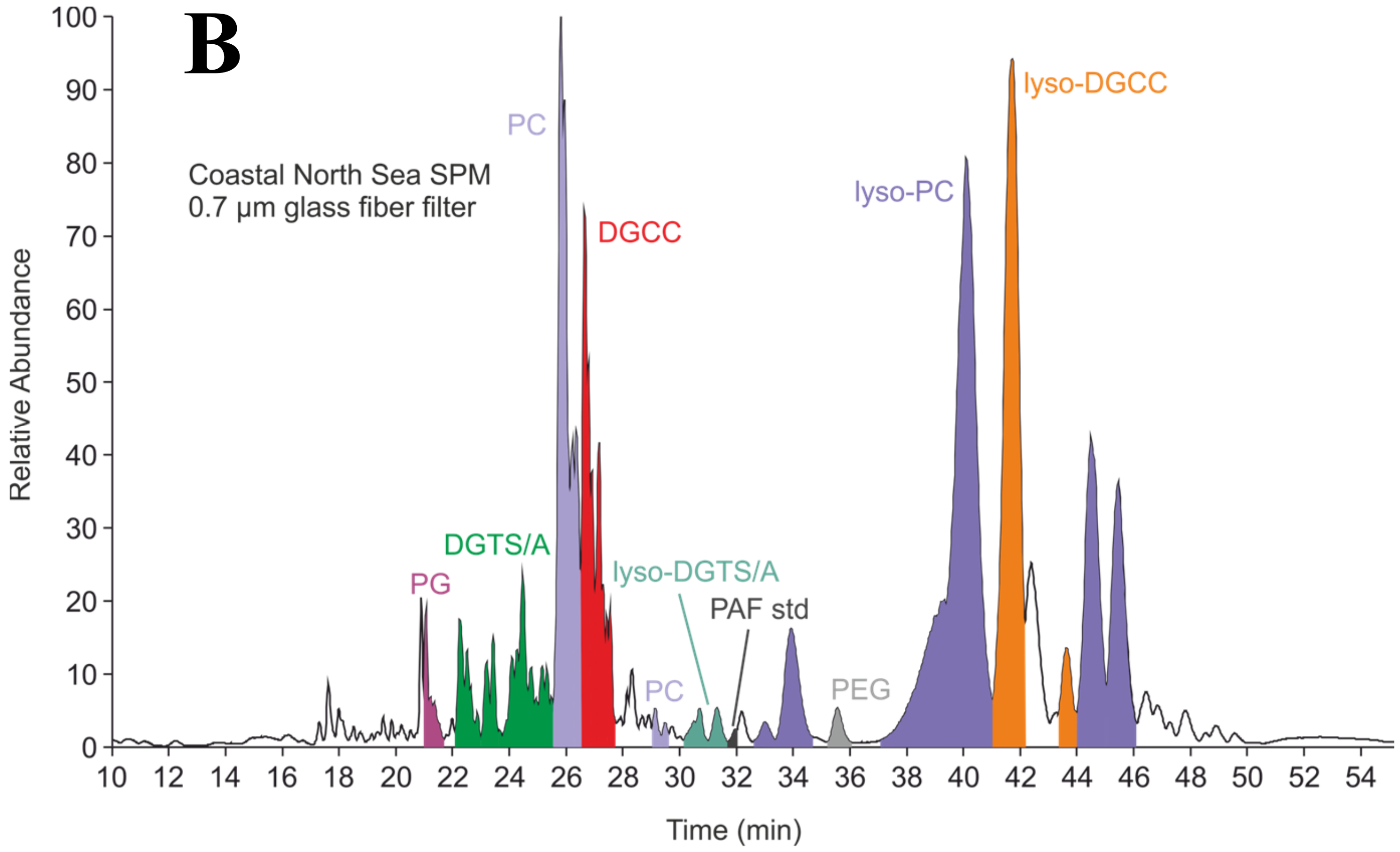

Figure S2. Partial base peak chromatogram of UHPLC-HRAM/MS analysis of IPLs in SPM. A) North Atlantic Ocean SPM collected at station 10 at 50 mbsl on 0.7 µm glass-fiber filters. B) coastal North Sea SPM. Abbreviations of peak labels: PC = phosphatidylcholine, PG = phosphatidylglycerol, SQDG = sulfoquinovosyldiacylglycerol, DGTS = diacylglyceryl-trimethyl-homoserine, DGTA = diacylglyceryl-trimethyl-alanine, DGCC = diacylglyceryl-carboxyhydroxymethyl-choline, DGDG = digalactosyldiacylglycerol, PEG = polyethylene glycol (contaminant), PAF = platelet activating factor (internal standard; 1-O-hexadecyl-2-acetyl-snglycero-3-phosphocholine). Lyso components are IPLs in which one of the fatty acids (FA) chains is lost. We were unable to distinguish between DGTA and DGTS betaine lipids using our analytical methods so this group is referred to as DGTS/A.

Table S1. 16S rRNA gene amplicon read counts of North Atlantic Ocean and coastal North Sea samples. After filtering and quality checks.

**Sample (station-depth)**	**Filter pore size (µm)**	**Number of reads**
NA, 12-5	0.7	335215
NA, 12-15	0.7	1295546
NA, 10-5	0.7	456206
NA, 10-9	0.7	701816
NA, 10-50	0.7	663975
NA, 10-200	0.7	516855
NA, 10-20	0.3	704703
NA, 10-50	0.3	670958
NA, 10-200	0.3	694986
NA, 8-5	0.7	833549
NA, 8-10	0.7	249967
NA, 8-52	0.7	628566
NA, 5-5	0.7	613496
NA, 5-40	0.7	329788
NA, 5-80	0.7	476872
NA, 4-5	0.7	681447
NA, 4-30	0.7	505062
NA, 4-83	0.7	775312
CNS, surface	0.7	475651

NA = North Atlantic Ocean, CNS = coastal North Sea.

Figure S3. Rarefaction curves showing the OTU richness (based on the 16S rRNA gene amplicon sequences, bacterial and archaeal OTUs) per sample. NA= North Atlantic Ocean, CNS = coastal North Sea, indicating the station and depth (meters below sea level) of sampling. The coastal North Sea sample filtered on the 0.3 µm filter was not included due to low abundance of reads.

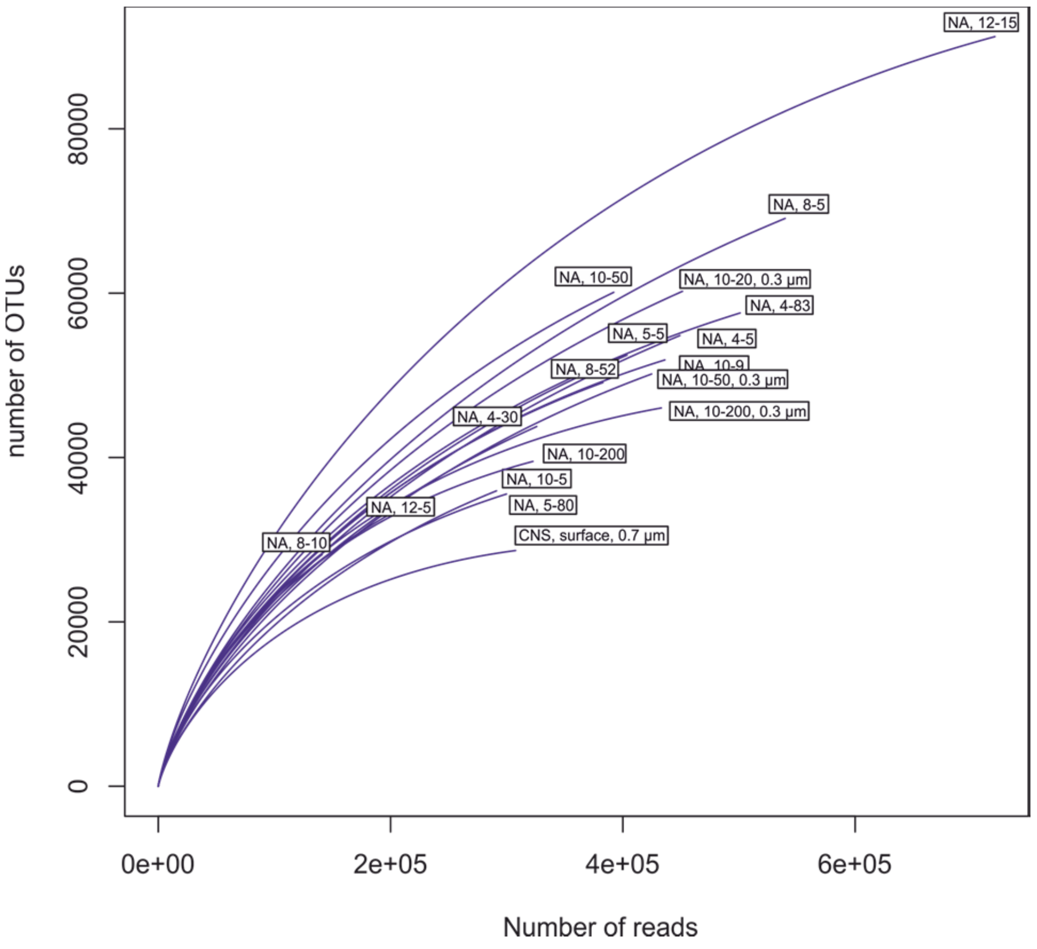

Figure S4. Heatmap of the abundance per archaeal group (based on the estimated 16S rRNA gene abundances per liter of seawater filtered) and the sum of the peak areas (l^{-1}) of the detected intact polar lipid (IPL-) glycerol dialkyl glycerol tetraethers (GDGTs). Green colors indicate a relative low abundance, red colors indicate a relative high abundance. NA= North Atlantic Ocean, CNS = coastal North Sea, indicating the station and depth (meters below sea level) of sampling.

Sample (station-depth)	Filter (pore size, µm)	Abundance per group (L^{-1}) MGI	MGII	MGIII	Peak areas (L^{-1}) IPL-GDGTs
NA, 12-5	0.7	1×10^7	5×10^7	1×10^7	8×10^5
NA, 12-15	0.7	9×10^6	3×10^7	7×10^6	5×10^5
NA, 10-5	0.7	1×10^6	2×10^7	2×10^6	0×10^0
NA, 10-9	0.7	1×10^5	1×10^7	8×10^5	0×10^0
NA, 10-50	0.7	2×10^6	5×10^8	4×10^7	0×10^0
NA, 10-200	0.3	9×10^6	1×10^7	2×10^6	8×10^5
NA, 10-20	0.3	7×10^4	7×10^6	2×10^6	0×10^0
NA, 10-50	0.3	1×10^5	2×10^7	3×10^6	0×10^0
NA, 10-200	0.3	8×10^7	7×10^7	1×10^7	2×10^6
NA, 8-5	0.7	3×10^5	1×10^7	9×10^5	0×10^0
NA, 8-10	0.7	5×10^5	2×10^7	2×10^6	0×10^0
NA, 8-52	0.7	3×10^7	3×10^8	2×10^7	1×10^6
NA, 5-5	0.7	3×10^5	1×10^7	8×10^5	0×10^0
NA, 5-40	0.7	2×10^5	1×10^7	2×10^6	0×10^0
NA, 5-80	0.7	1×10^8	2×10^8	2×10^7	2×10^6
NA, 4-5	0.7	2×10^5	1×10^7	1×10^6	0×10^0
NA, 4-30	0.7	8×10^4	7×10^6	1×10^6	0×10^0
NA, 4-83	0.7	5×10^7	2×10^8	1×10^7	2×10^6
CNS, surface	0.7	7×10^6	1×10^9	0×10^0	0×10^0

Table S1. PCR primers, efficiency and correlation coefficient of the quantitative QPCR assays.

QPCR (gene)	Primers	Reference	Efficiency	R^2
Archaeal 16S rRNA	Parch519F/ARC915R	Pitcher et al.[10]	91.8	0.94
Archaeal 16S rRNA	Parch519F/ARC915R	Pitcher et al.[10]	91.4	1

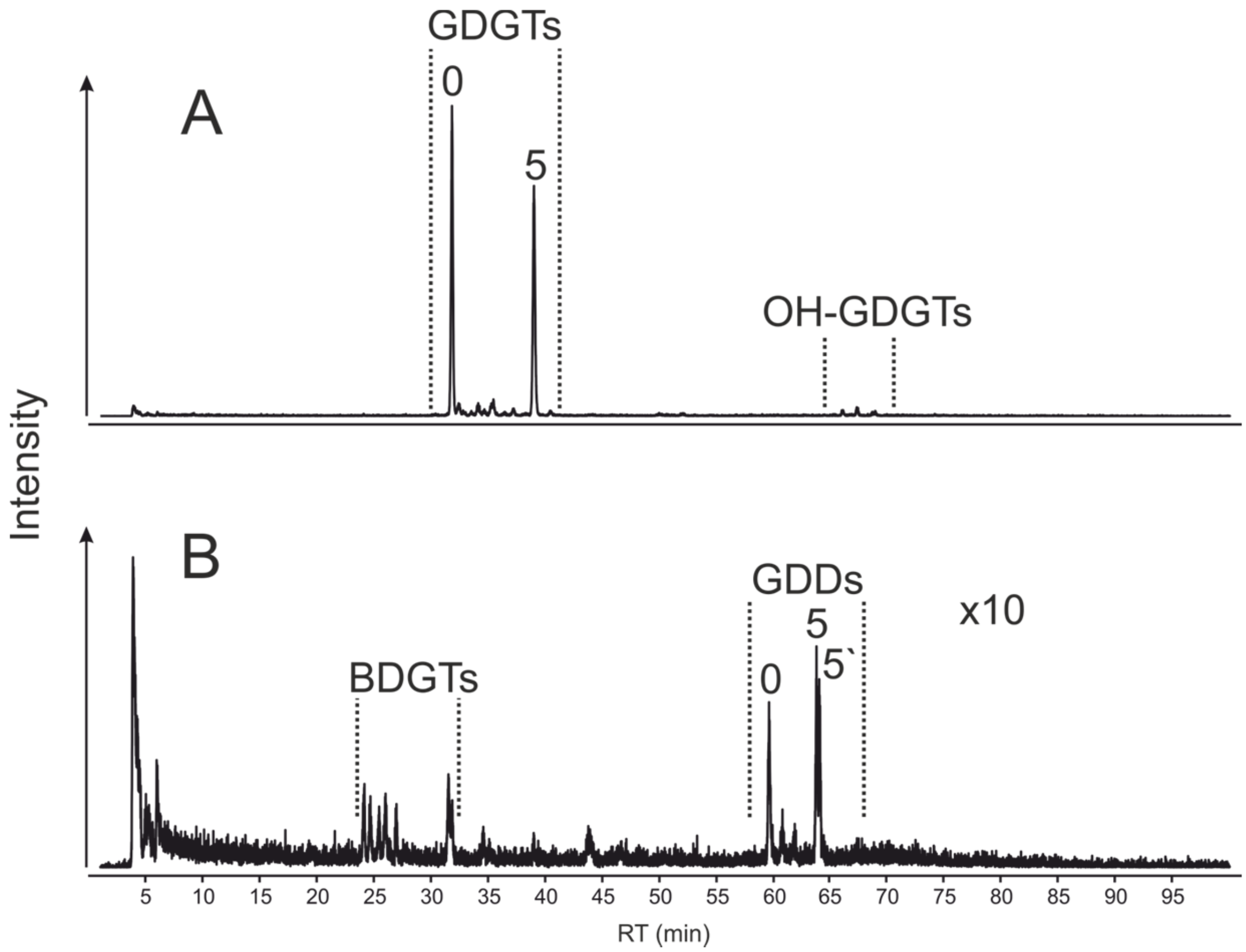

Figure S5. UHPLC-ToFMS analysis of the acid hydrolyzed Bligh and Dyer extract of SPM from the coastal North Sea sampled with a 0.7 μm glass-fiber filter. (A) Summed mass chromatograms (within 10 ppm mass accuracy) of the [M+H]+ ions of GDGTs 0-8 and hydroxyl GDGTs (OH-GDGTs 0-8). (B) Summed mass chromatograms (within 10 ppm mass accuracy) of the [M+H]+ ions of butanetriol dibiphytanyl glycerol tetraethers (BDGTs 0-8) and glycerol dialkanol diethers (GDDs 0-8), within 10 ppm mass accuracy. The mass chromatogram in panel (B) is 10x magnified compared to that in panel (A). Labels of peaks indicate the number of cyclic moieties, where the peaks labeled '5' are the analogues of crenarchaeol with four cyclopentyl and one cyclohexyl moiety. RT = retention time.
